# Gaussian Mixture Models and Model Selection for [18F] Fluorodeoxyglucose Positron Emission Tomography Classification in Alzheimer’s Disease

**DOI:** 10.1371/journal.pone.0122731

**Published:** 2015-04-28

**Authors:** Rui Li, Robert Perneczky, Igor Yakushev, Stefan Förster, Alexander Kurz, Alexander Drzezga, Stefan Kramer, Alzheimer’s Disease Neuroimaging Initiative

**Affiliations:** 1 Institut für Informatik/I12, Technische Universität München, Garching bei München, Germany; 2 Klinik und Poliklinik für Psychiatrie und Psychotherapie, Technische Universität München, München, Germany; 3 Nuklearmedizinische Klinik, Technische Universität München, München, Germany; 4 Klinik und Poliklinik für Nuklearmedizin, Universität zu Köln, Köln, Germany; 5 Institut für Informatik, Johannes Gutenberg-Universität Mainz, Mainz, Germany; University of Cambridge, UNITED KINGDOM

## Abstract

We present a method to discover discriminative brain metabolism patterns in [18F] fluorodeoxyglucose positron emission tomography (PET) scans, facilitating the clinical diagnosis of Alzheimer’s disease. In the work, the term “pattern” stands for a certain brain region that characterizes a target group of patients and can be used for a classification as well as interpretation purposes. Thus, it can be understood as a so-called “region of interest (ROI)”. In the literature, an ROI is often found by a given brain atlas that defines a number of brain regions, which corresponds to an anatomical approach. The present work introduces a semi-data-driven approach that is based on learning the characteristics of the given data, given some prior anatomical knowledge. A Gaussian Mixture Model (GMM) and model selection are combined to return a clustering of voxels that may serve for the definition of ROIs. Experiments on both an in-house dataset and data of the Alzheimer’s Disease Neuroimaging Initiative (ADNI) suggest that the proposed approach arrives at a better diagnosis than a merely anatomical approach or conventional statistical hypothesis testing.

## Introduction

Alzheimer’s disease (AD) is a progressive, degenerative and incurable disease of the brain and the main cause of dementia. The number of people suffering from dementia is expected to grow rapidly in the next decades due to increasing life expectancy [1], which will have a major negative impact on healthcare systems worldwide. Despite technological progress, the ante mortem diagnosis of AD is still based on clinical grounds, with biomarkers such as cerebrospinal fluid (CSF) proteins and neuroimaging procedures providing supporting information.

Positron emission tomography with [18F] fluorodeoxyglucose (FDG-PET) has been widely applied to assist the diagnosis of AD [2]. Accordingly, FDG-PET was recommended as a diagnostic marker by recently proposed guidelines [3]. Visual examination of images may be error-prone, thus computer-aided diagnosis (CAD) has attracted many researchers’ interest to diagnose dementia based on medical imaging [4, 5].

One technique often used for CAD in this area, automatic image classification, remains a subject of intensive research. Overall, however, more studies have been conducted on magnetic resonance imaging (MRI) than on PET images. Regional brain atrophy, especially of the mediotemporal lobe, is a typical feature of AD, which can be reliably identified by MRI [6–8], and is therefore a useful imaging biomarker. For example, a recent study [9] compared ten distinct MRI classification approaches using 509 subjects of the Alzheimer’s Disease Neuroimaging Initiative (ADNI), investigating the differentiation between different groups of individuals including normal controls (NC) against patients with AD. Another work [10] presented an ensemble approach to combining a number of weak classifiers for classification. This local patch-based (a patch is understood as some small region) subspace ensemble method builds individual classifiers based on various subsets of local patches and then combines them for a better classification.

In recent years, multi-modality classification has been shown to be an attractive research area in AD research. A pairwise similarity measure derived from random forests was proposed as a multi-modality classification framework [11]. In this study, FDG-PET, MR, CSF biomarker and categorical genetic data were employed for the classification. The results indicated that joint information is superior to any individual modality on its own. A similar approach, stacked multi-view learning [12], showed that diverse information can benefit the classification substantially, bringing together neuropsychological tests, PET scan images and demographical variables. In another work, MRI data, PET data and CSF biomarkers were used to construct a kernel matrix, and a combined kernel was produced for the final classification [13]. This method allows combining heterogeneous data and permits different weights for various data modalities. The results show high AD classification accuracy, even in very early clinical stages (i.e., for mild cognitive impairment, MCI).

We also review some related work on model selection based on PET images, in areas other than dementia research. In a PET volume classification study [14], BIC was applied to select the optimal number of classes for each PET scan. In this work, the BIC values gradually reached a steady state, such that the optimal number of classes could easily be chosen. Another work [15] employed AIC to assess the different predictive models, investigating the overall survival in a phase II clinical trial of a targeted therapy. The authors reported that the highest prognostic value appeared with the lowest AIC value, which suggested that AIC can be a guideline in choosing a desired model. In a proton therapy research study [16], the AIC was used to determine the most appropriate model for the FDG uptake dose response for each patient. A compound-B based PET kinetic modeling study [17] used AIC to ensure a good kinetic parameter setting. In another study [18], however, both AIC and BIC did not perform reliably for realistic 3D dynamic PET images. The authors assumed that the reason for this may be that AIC and BIC are model dependent so that the specified probability distribution function was not suitable for realistic 3D dynamic PET images. Although AIC and BIC are frequently applied model selection techniques in neuroscience, their application to PET scans for the purpose of AD diagnosis is rarely studied. Hence, this work studies their usefulness in combination with the proposed GMM approach in dementia research.

The goal of the present research is to explore the diagnostic usefulness of a novel CAD-based approach for FDG-PET data. From a technical point of view, the neuroimaging community frequently employs Statistical Parametric Mapping (SPM) [19] in a univariate statistical testing approach to localizing voxels that are discriminative for different groups. This approach suffers from considering voxels independently, which is not taking into account correlations between neighbouring voxels. In contrast to such a univariate approach, multivariate methods were proposed to learn the patterns in data from a higher perspective. For example, principal component analysis (PCA) is used to extract features, which are then fed into a classifier [20]. Another work [21] used PCA to analyze FDG-PET in amnestic MCI and illustrated some interesting findings using the principal components. It is worth mentioning that PCA essentially transforms the original features into another feature space, which is different from the method proposed in the following.

A Region of Interest (ROI) based method was developed to derive features from PET images [22, 23]. A Gaussian Mixture Model (GMM) models the difference between controls and patients with AD, where the number of Gaussians (*K*) was fixed to 64, which can be a drawback since 64 may not be the optimal value. In this paper, the proposed approach is able to choose the optimal *K* by a model selection technique. Because the correct diagnosis of dementia is also of practical interest, we therefore propose a novel CAD approach that discriminates between NC, MCI and full-blown AD.

## Materials and Methods

### Ethics Statement

Each patient in the in-house dataset, gave written informed consent to participate in the study. Patients' names were anonymized in the database. The study is allowed to be conducted by Technische Universität München (TUM). The protocols were submitted to appropriate Boards and their written unconditional approval obtained and submitted to Regulatory Affairs at the Alzheimer’s Disease Neuroimaging Initiative Coordinating Center (ADNI-CC) prior to commencement of the study. Further information about ADNI can be obtained from www.adni-info.org.

### Data Acquisition

Experiments were performed on two independent datasets. One is from the publicly available ADNI database (http://adni.loni.usc.edu/) and the other is an in-house dataset of patients and controls recruited at the memory outpatient unit of the Department of Psychiatry at Technische Universität München. The above datasets are in the following referred to as ADNI and TUM, respectively. ADNI has a large pool of PET (co-registered, averaged) images, which have been acquired on various scanners using different imaging parameters. To eliminate the bias of these factors, we selected images that have been obtained using the same scanner as well as the same parameters, such as the number of slices. The patient information and the PET scanner type are summarized in Table 1. Further details about the ADNI recruitment procedures are provided in the acknowledgments.

**Table 1:** Participant characteristics and scanner type.

		Sex (male: female)	Age (mean±SD)	MMSE (mean±SD)	Scanner Type
ADNI	NC	30 (21:9)	74±5	28.6±1.35	Siemens/CTI
	MCI	29 (23:6)	74±6	27.4±1.68	Siemens/CTI
	AD	25 (15:10)	72±6	23.2±2.23	Siemens/CTI
TUM	NC	16 (7: 9)	66±6	29.3±0.70	Siemens Ecat HR Plus
	MCI	30 (16:14)	69±7	26.3±2.41	Siemens Ecat HR Plus
	AD	30 (18:12)	69±8	21.5±5.23	Siemens Ecat HR Plus

NC: normal control, MCI: mild cognitive impairment, AD: Alzheimer’s disease, TUM: Technische Universität München dataset, ADNI: Alzheimer’s Disease Neuroimaging Initiative dataset, MMSE: Mini-Mental-State Examination.

Prior to their use for image analysis, PET images had to undergo two pre-processing steps: spatial normalization and smoothing (kernel size [8 8 8] mm), which were achieved by SPM5. The spatial normalization ensures that the processed image is of the size 91×109×91, which is in accordance with Anatomical Automatic Labeling (AAL) [24]. The final step is the intensity normalization that was done by dividing each voxel by the mean intensity value averaged over all brain voxels (grand mean normalization, the non-brain voxels surrounding the brain were excluded). The second intensity normalization method is called pSMC (primary sensorimotor cortex) and was reported to be advantageous in a study [25]. Anatomically, the “Precentral_L, Precentral_R, Postcentral_L and Postcentral_R” regions in the AAL brain template can be used as the primary sensorimotor cortex.

### Gaussian Mixture Model and Model Selection (GMM+MS)

GMM [26] is a parametric density estimation approach that assumes the data is generated by more than one Gaussian distribution. It can cluster a point by assigning the class label to the Gaussian that contributes the largest probability. Given a vector (data point) *x*,a GMM is defined as:

$$p(x|\theta )=\sum_{k=1}^{K}\pi_{k}ℵ(x|\mu_{k},\Sigma_{k})$$ (1)

where *μ*_*k*,_Σ_*k*_ and and *π*_*k*_ are the mean, covariance and mixing proportion respectively. In addition, $\sum_{k=1}^{K}\pi_{k}=1,$ *π*_*k*_ ≥ 0 and *θ* = {*μ*_*k*_,Σ_*k*_,*π*_*k*_}. ℵ denotes the *D*-dimensional Gaussian distribution:

$$ℵ(X|\mu ,\Sigma )=\frac{1}{{\left(2\pi \right)}^{\frac{D}{2}}{\left|\Sigma \right|}^{\frac{1}{2}}}\exp\left(-\frac{1}{2}{\left(X-\mu \right)}^{T}\Sigma^{-1}(X-\mu )\right).$$ (2)

Expectation Maximization (EM) [27] is usually used to solve a GMM by the following steps: E-step: estimate the posterior probability $p_{ik}^{t}$ at *t* iteration as:

$$p_{ik}^{t}=\frac{\pi_{k}^{t}p(x_{i}|\mu_{k}^{t},\Sigma_{k}^{t})}{\sum_{k=1}^{K}\pi_{k}^{t}p(x_{i}|\mu_{k}^{t},\Sigma_{k}^{t})}.$$ (3)

M-step: update the parameters *μ*_*k*,_Σ_*k*_ and *π*_*k*_ at *t*+1 iteration based on the probabilities from the E-step:

$$\pi_{k}^{t+1}=\frac{1}{N}\sum_{i=1}^{N}p_{ik}^{t},$$ (4)

$$\mu_{k}^{t+1}=\frac{\sum_{i=1}^{N}p_{ik}^{t}x_{i}}{\sum_{i=1}^{N}p_{ik}^{t}},$$ (5)

$$\sum_{k}^{t+1}=\frac{\sum_{k=1}^{K}p_{ik}^{t}(x_{i}-\mu_{k}^{t}){\left(x_{i}-\mu_{k}^{t}\right)}^{T}}{\sum_{i=1}^{N}p_{ik}^{t}}.$$ (6)

EM is guaranteed to converge, so that a locally optimal solution is always assured. However, one big concern in GMMs is the number of components/clusters (*K*) that must be defined in advance, which can be a hard task. We employ the Bayesian Information Criterion (BIC) [28] to determine this parameter automatically. The BIC is frequently used for model selection, since it considers a trade-off between model fitting and model complexity. By adding a penalty term for the number of parameters in the model, the BIC can alleviate the problem of overfitting, which can be caused by increasing the likelihood by just adding parameters to the model. The BIC has the form:

BIC=−2log(likelihood)+log(N)P, (7)

where *P* is the number of free parameters to be estimated in the model and *N* is the total number of data points. In a multivariate Gaussian, the number of free parameter is (*KD*(*D*+3))/2+*K*-1,due to *K*-1 mixing proportions to decide, *KD* mean values, and (*KD*(*D*+1))/2 free parameters in the covariance matrix. The model with the lowest value of BIC is selected as the desired model. Aside from BIC, the Akaike Information Criterion (AIC) [29] is also a common method for model selection.

AIC=−2log(likelihood)+2P, (8)

The log-likelihood (*L*) of the GMM can be inferred as:

$$\begin{array}{l} L(X|\pi ,\mu ,\Sigma )=\sum_{n=1}^{N}\log\left\{\sum_{k=1}^{K}\pi_{k}ℵ(x_{n}|\mu_{k},\Sigma_{k})\right\}\\ \;=\sum_{n=1}^{N}\log\left\{\sum_{k=1}^{K}A_{k}\right\},\;A_{k}\text{ is a multivariate Gaussian}ℵ(X|\mu ,\Sigma )\\ \;=\sum_{n=1}^{N}\left\{\log A_{m}+\log\left(\sum_{k=1}^{K}\exp(\log A_{k}-\log A_{m})\right)\right\},\text{ log-sum-exp trick}\\ \;let\;A_{m}=\max\left\{\pi_{i}ℵ(x_{n}|\mu_{i},\Sigma_{i})\right\},\;i\in 1,\ldots K\\ \;=\sum_{n=1}^{N}\left\{\log\pi_{m}-\frac{D}{2}\log2\pi -\frac{1}{2}\log\left|\Sigma_{m}\right|-\frac{1}{2}\sum_{n=1}^{N}{\left(x_{n}^{m}-\mu_{m}\right)}^{T}\Sigma_{m}^{-1}(x_{n}^{m}-\mu_{m})\right\}+\cdots \\ \;+\sum_{n=1}^{N}\left\{\log\left(\sum_{k=1}^{K}\exp(\log A_{k}-\log A_{m})\right)\right\}, \end{array}$$ (9)

where the “log-sum-exp” trick [30] is a method for avoiding numeric underflow and thus can improve the numeric stability when computing the BIC in a GMM scenario. Continue writing out the Eq 9, we can compute the log-likelihood of the GMM, which is needed for computing Eq 7 and Eq 8.

It can be seen that AIC (the lower, the better) has a lower penalty for model complexity, because log(*N*)*P* is usually much larger than 2*P*. Because both AIC and BIC consist of two terms, the final scores weight the relevance of these two terms. In AIC, the term 2*P* does not contribute much to the final score as opposed to -2log(likelihood). However, log(*N*)*P* increases much faster when the model becomes more complex (more clusters), thus the resulting BIC score stops growing as the number of clusters increases. From Eqs 7 and 8, we see that AIC and BIC suggest different quantities. Thus, they may yield different model selection results. In the present study, we conduct experiments using both AIC and BIC, and report more results on BIC. In the following, we review some reported studies comparing BIC with AIC. Our experiments show that AIC and BIC yield comparable results.

**Fig 1:**
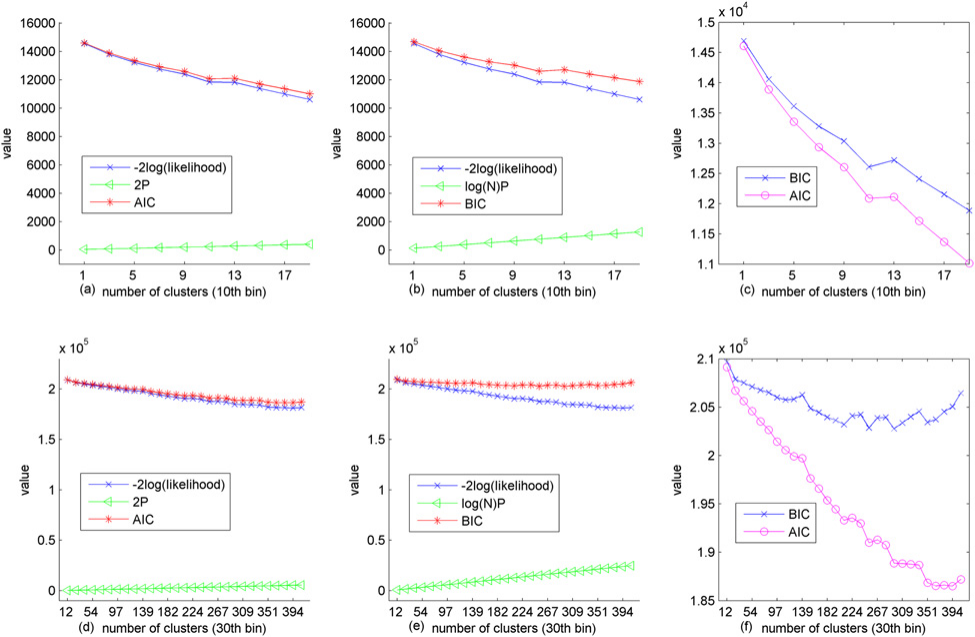
BIC and AIC score displayed for the 10^th^ (top) and 30^th^ (bottom) bin in 50 bin. Results are obtained by using pSMC normalization of the ADNI dataset. The AIC (red) and BIC (red) values are simply -2log(likelihood) (blue) plus 2*P* (green) and log(*N*)*P* (green), respectively. Refer to Eq 7 and Eq 8 for the definition of AIC and BIC. (a), (b) (d) and (f) show the AIC and BIC along with their components' value. (c) and (f) offers a direct comparison between AIC and BIC. The 10^th^ bin contains 586 voxels, hence it is a small sample, in contrast to the large sample of the 30^th^ bin containing 12,414 voxels. Note that the term “sample” in this context does not mean the number of patients, but the number of voxels to be clustered.

A recent study [31] reveals a similar observation that illustrates model selection discrepancy between AIC and BIC, preferring BIC to AIC for the sake of favoring simpler models. In addition, another work [32] explicitly states that “AIC has been shown to perform well at selecting the true number of factors when it exists, but only at small sample size *N*. BIC has been found to outperform AIC in recovering the true *K* (number of clusters)”. Particularly, we observe that AIC and BIC tend to agree with each other when the number of voxels (sample size) is small (i.e., 586, cf. Fig 1). As the number of voxels increases to 12,414, BIC reaches the lowest value much earlier than AIC, which is illustrated in Fig 1. Thus, BIC is more appropriate when the number of voxels is large (greater than 3500 may mean “large”, refer to work [33] to avoid a too complex model. As for the plot (c) in Fig 1, it can be seen that the AIC and BIC continues dropping as the number of clusters increases. Thus, one may conclude that the more clusters, the better in the small sample case. However, we can, at least, observe a dip if we test a larger number of clusters. Because we intentionally impose that a cluster should at least contain 30 voxels to avoid trivial clusters, more clusters are not necessary to be tested (refer to Section Parameters in Model).

Another study shows that BIC performs better than AIC in both small and large sample size cases, claiming that AIC lacks the appropriate penalty to prevent overfitting (see Table 2 of the original reference [34]).

The difference between our BIC models is greater than 10 (see plot (c) and (f) in Fig 1), thus it suggests strong evidence (cf. Table 2) that the model difference is meaningful according to work on Bayes factors [35].

**Table 2:** Grade of evidence of the BIC difference [35].

BIC difference	Evidence
0–2	weak
2–6	positive
6–10	strong
>10	very strong

**Fig 2:**
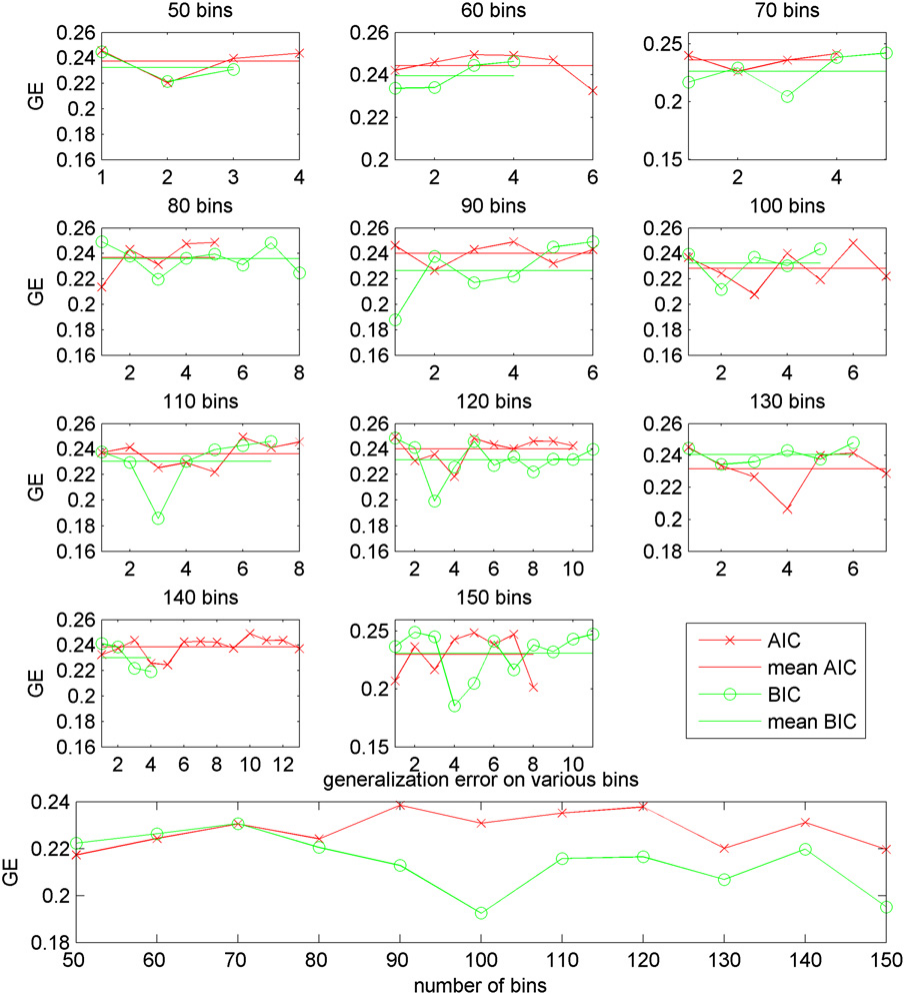
Generalization error (GE) by BIC and AIC using pSMC normalization of MCI versus AD on the ADNI dataset. The generalization error (equivalently, 1-accuracy) is computed based on features (mean and standard deviation value) extracted from the clusters in the individual bin (one bin among 50 bins, for example) via 10 times 10-fold cross-validation. For example, a green point can represent the classification GE using *n-*th bin when we divide the whole brain voxels into 50 bins. The plotted points are the bins that yield GE < 0.25, and these bins are highly predictive, thus contribute to the final classification results. The x-axis represents only the number of points, implying no ordering. The horizontal green line and red line are the mean values computed from green circles and red crosses, respectively. The bottom plot illustrates the generalization error from dividing brain voxels into 50 bins till 150 bins after collecting top informative features from the individual bins.

To empirically show the difference between AIC and BIC in this study, we show the generalization error (GE, equivalently, 1-accuracy) demonstrated by Fig 2. From the 50 bins to 150 bins, we observe that BIC outperforms AIC on 8 cases (50, 60, 70, 80, 90, 110, 120, 140 bins) in terms of mean generalization error. BIC often yields a certain bin that has the lowest GE, for example, in 60 bins, 70 bins, 90 bins, 110 bins, 120 bins, 140 bins and 150 bins. When we perform the classification using all features extracted on all bins together (details are explained in Section GMM+MS on 3D PET images), we see that BIC shows lower GE than AIC demonstrated by the bottom plot of Fig 2. The reason for this is that selected features (on all bins) by BIC contain more discriminative information than the features by AIC, which complies with the results of MCI against AD using pSMC normalization using ADNI dataset. However, AIC shows better performance than BIC in some other cases, such as MCI against AD using TUM dataset. In summary, AIC and BIC suggest generally comparable results across the two datasets in different classification cases.

### GMM+MS on 3D PET Images

We employ a clustering method (GMM) to group brain voxels into small regions that exhibit both high similar intensity and geometric affinity. A PET image can be viewed as three dimensional (3D) spatial data along with one extra dimension that represents the intensity of each voxel. Thus, a voxel is denoted by a 4-tuple (*x*,*y*,*z*,*I*)∈ℜ^4^, where *x*,*y*,*z* are the spatial coordinates and *I* is the intensity value. We used NC PET images as reference images to obtain the clustering results and used these clusters to extract the features from the NC, MCI and AD groups. Note that the method is applied on the AAL (gray matter voxels of MNI space) defined voxels, which constitute the gray matter in the brain. The mean intensity and standard deviation of each cluster are subsequently defined as features. To ensure the clusters have similar intensity values and are geometrically connected, we first group the original voxels into a certain number (e.g., 100) of bins of equally sized intensity ranges, and then cluster each bin by the introduced methods based only on the spatial information, i.e., the *x*,*y*,*z* coordinates. A bin is a data interval that is described by a statistical histogram. The data falling into the same bin are from a certain value interval, such that the data within the same bin are similar in their values. Theoretically, it is hard to find the most appropriate number of bins in advance, thus we tested different numbers from 50 to 150 with step size 10, i.e., 50, 60, …, 150. The best one can be chosen by a cross-validation on the training data. In practice, cross-validation is repeated in a way that the division into sets is identical for the various bin sizes. Given the training data, we can further split them into sub-training and sub-test data, which are used to train and evaluate the model respectively. Evaluating the model using the sub-test data gives a predictive accuracy. The yielded highest accuracy of a certain bin corresponds to the most appropriate number of bins. Once the number of bins is determined in this way, the same training procedure can be applied to the whole training data to maximize the use of present training data. Therefore only the training data is used to set the optimal parameters in the experiments. The workflow of the proposed method is summarized in Table 3.

**Table 3:** Workflow of proposed GMM+MS clustering method on 3D PET images.

1.	Stratified 10-fold cross-validation
2.	*Training phase (9 folds)*
	Run different bins 50, 60, 70, …, 150. Split training data into disjoint sub-training and sub-test data.
	i. Given a Mean NC image, divide the AAL defined voxels into the specified number of bins, such as 50. Mean NC is averaged over all NC images.
	ii. For each bin, run GMM+MS method to yield the clusters.
	iii. Collect all the resulting clusters from all the bins.
	iv. Given a NC, MCI or AD image from the pool of sub-training data, compute the mean (*μ*) and standard deviation (σ) of the voxels in each cluster using the provided spatial information, i.e., *x*,*y*,*z* of the cluster.
	v. The image can then be represented as a feature vector of the means (*μ*) and standard deviations (σ).
	vi. Build SVM model using only the sub-training data, and the predictive accuracy is computed for the sub-test data using the model. (The model is trained on MCI and AD sub-training data, if the classification is MCI against AD.)
	Collect the computed accuracy from all bins.
3.	The resulting clusters correspond to the bin with the highest accuracy are used as the most appropriate clusters for NC, MCI and AD images.
4.	*Test phase (remaining 1 fold)*
	i. Construct the features for both training and test images described as the steps of “iv and v”.
	ii. Build SVM model using the training data (9 folds), and obtain the results using the remaining test data (1 fold).

**Fig 3:**
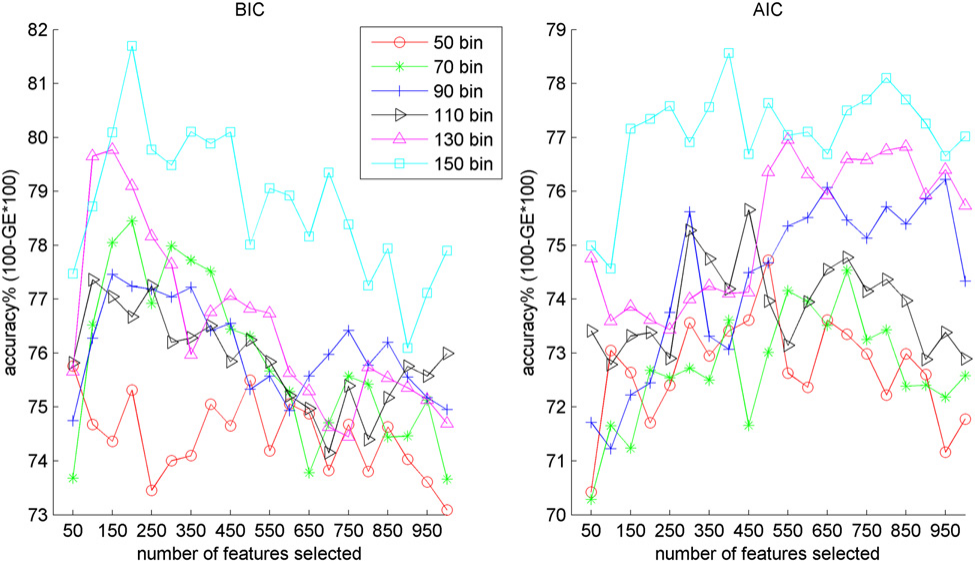
Number of features selected versus the classification accuracy using pSMC normalization. Results are obtained by using the ADNI dataset on MCI against AD. Accuracy of BIC tend to drop after selecting 150 features, and AIC tends to drop after 500.

If there are 1000 clusters formed using GMM+MS, then the image can be represented as a 2000 (1000*μ* and 1000σ) dimensional vector. Generally, not all of these features are informative, thus we applied a feature selection technique [36] to choose the most discriminative ones for building the model. In this study, we empirically used the top-150 most informative features for learning the model. From Fig 3 of BIC, we observe that the classification accuracy increases in the beginning, but drops after selecting too many features. 150 features appear to provide sufficient classification relevant information, and more features may hamper the classifier's performance due to the well-known curse of dimensionality [26]. As for AIC, top-400 features were selected to perform the experimental comparison. AIC needs more features than BIC, which may be the reason why AIC divides voxels into more clusters so that the discriminative information are spread over many clusters. It should be pointed out that the feature selection [37] and the model building steps only used information from the training set: no information from the test set is used at any point in time (in other words, no information *leakage* from the test set to the training set has occurred).

A support vector machine (SVM) was used to build the final classification model, which is trained on the training data. The SVM has been shown to perform well in a variety of applications, thus it was chosen to be the classifier in this study. A tutorial [38] offers a good introduction to the SVM. Apart from the SVM, other classification methods could be used, such as Random Forests, Naïve Bayes, and others. We do not attempt to compare the proposed method with SVMs, we rather use a SVM as a classifier in the method. The suggested method aims at extracting useful features from brain voxels, whereas SVMs are a classification method based on input features (voxels).

In terms of running time, it costs roughly 15 hours to cluster the mean NC image from 50 bins to 150 bins. It takes only a few seconds to extract the features of a given PET scan after having the clusters. Therefore, the proposed approach is very efficient once the clusters are derived, since extracting features from new images is fast. The code is implemented in MATLAB and runs on a machine with Intel(R) Core i7-3632QM CPU @2.20 GHz, 8GB of memory. In addition, the LIBSVM [40] package provides a fast classification, once the features are constructed.

### Compared Methods

AAL approach: AAL [24] defines 116 brain regions, and we extracted the mean and standard deviation from each of these regions as features. Thus, in total each image is represented by a 232-dimensional feature vector.

T-test: This hypothesis testing based method uses voxel-wise t-test and is widely applied in neuroscience studies. A t-test based method, for example, was tested as a method for feature selection with respect to predictive accuracy recently [39]. If the *p*-value is lower than a pre-defined threshold, e.g., 0.001, then this voxel is regarded as an indicator voxel for two groups of individuals. The null hypothesis is that the voxels in the two groups come from a population where the means of the two groups are the same. Therefore, a *p*-value lower than 0.001 rejects the null hypothesis, i.e., the two groups have different means. Hence, this voxel can be an indicator voxel representing group difference. We performed a two-tailed t-test on each of the voxels defined in an AAL region without multiple comparison correction, with a threshold set to 0.001. Finally, the top-150 voxels with the lowest *p*-value were chosen for learning, to be in line with our proposed method.

### Test Protocol

The experiments were performed on two datasets (ADNI and TUM, cf. Table 1) independently. For each of the datasets, we trained the model based on the training data, evaluating the model using the held-out test data. The training and test data were divided using 10-fold cross-validation. In statistics, 10-fold cross-validation is commonly applied as an approach to testing a predictive model. In 10-fold cross-validation, technically, the original dataset is roughly divided into ten subsets, and each time we select nine subsets for training the model and the remaining one for testing. This procedure is repeated ten times, assuring that each subset is tested exactly once and employed nine times for training. The ten results are then averaged to produce a single estimate. (In case of very small data sets, results are aggregated by instance and not by test set.) In the two-class classification case, each subset contains roughly the same proportion of samples from the two classes, which is called *stratification*. For MCI versus AD in the TUM dataset (30 MCI, 30 AD), for instance, each subset consists of six samples with three from the MCI and three from the AD group. Each time 54 samples are employed for training and six for testing. After repeating the procedure ten times, we compute the mean value from all ten runs as the final result. We used the implementation of “crossvalind” function in MATLAB 2010 (R2010a) to achieve the stratified 10-fold cross-validation.

### GMM+MS in Summary

To sum up, we tested different numbers of bins (50, 60 to 150 with a step size of 10) to decide how many bins the whole brain voxels should be divided into. For example, if the brain voxels are divided into 50 bins, we run the GMM algorithm on each of the 50 bins. The BIC suggests the optimal number of clusters in each bin. The yielded clusters from the 50 bins are then collected as the final clusters in the whole brain. The mean and standard deviation of each cluster (given *x*, *y*, and *z* coordinates) are extracted as the feature values representing a PET scan. Finally, every PET scan can be represented by a vector. A 10-fold cross-validation is then used to train an SVM and test the classification performance based on the vectors. Table 3 further explains the procedure of the method applied to PET scans.

## Results

### Parameters in Model

**Fig 4:**
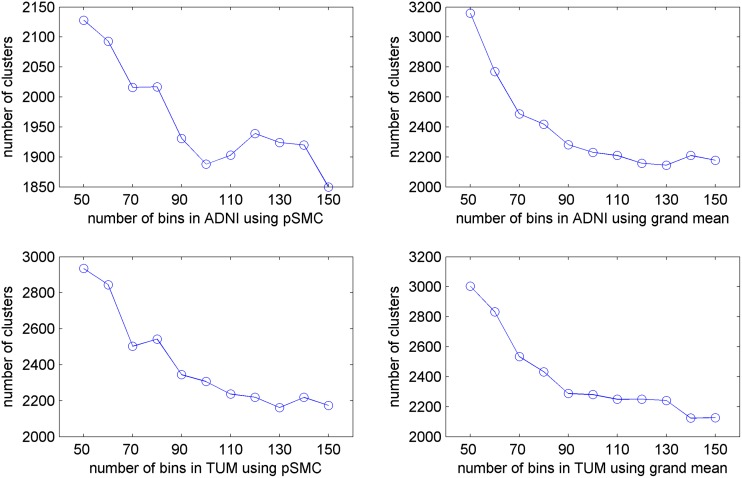
Relation between the number of clusters and the number of bins on ADNI and TUM datasets. Experiments are conducted on the normal control PET scans.

The proposed method is able to find the optimal number of clusters by comparing the BIC score computed from different models. Thus, a number of different model selections must be performed. The simplest way to determine the number of clusters in a model is to let the cluster number be equal to the number of voxels in each studied bin (a voxel value interval). However, this is not only computationally expensive, but we may also end up with clusters that include too many (too rough) or too few (too trivial) voxels. To this end, we intentionally defined a priori that the resulting clusters should have 1000 voxels at most and 30 voxels at least. Then the maximal number of clusters in a bin can be calculated as Cmax = #(voxels in a bin)/1000, and the minimal is Cmin = #(voxels in a bin)/30.The numbers between the Cmax and Cmin are used in these model selections. Fig 4 depicts that the number of derived clusters, in general, decreases as the number of bins increases. However, the sharp drop appears in the beginning and the curve then gradually reaches a nearly stable state until 150.

### Performance Comparison

Table 4 shows that the GMM+MS method performs much better than the AAL and t-test methods on the task of discriminating MCI from AD. In particular, GMM+MS is statistically significantly better than the t-test method. Specifically, regarding pSMC, the accuracy gain is 12.9% (80.2%– 67.3%) with a *p*-value of 0.017 (calculated using the test suggested by Bouckaert et al., [41]), and the specificity gain is 0.19 (0.80–0.61) with a borderline *p*-value of 0.066. Regarding the grand mean normalization method, the accuracy, AUC, sensitivity and specificity all show better results than the AAL and t-test approaches. As for NC versus AD, the three methods perform equally well, which may due to the fact that the most essential discriminative information can be easily identified by all of them. As a result, further improvement hardly can be achieved. The t-test approach reveals a slightly better result on NC versus MCI using pSMC, whereas it shows similar performance using grand mean normalization. However, the opposite is true on the TUM dataset (cf. Table 5), which suggests that GMM+MS is much better than the other two methods. Again, a comparable performance is shown for NC versus AD. Regarding MCI versus AD, the grand mean still reveals improved results, and pSMC shows comparable performance. As for the comparison between AIC and BIC, there is no significant difference revealed by the results, cf. Tables 4 and 5. A possible explanation is that both AIC and BIC can discover discriminative clusters sufficiently well, despite the fact that AIC favors a more complex model and BIC tends to choose a simpler one.

**Table 4:** Result summary of three different methods on ADNI dataset.

			Accuracy %	AUC	Sensitivity	Specificity
MCI vs. AD	P	GMM+MS	80.2* (78.3)	**0.85 (0.82)**	**0.80 (0.77)**	**0.80 (0.79)**
		AAL	74.2	0.81	0.75	0.74
		t-test	67.3	0.79	0.72	0.61
	G	GMM+MS	**77.1 (78.1)**	**0.83 (0.83)**	**0.85 (0.78)**	**0.68 (0.77)**
		AAL	73.2	0.80	0.77	0.68
		t-test	69.5	0.81	0.76	0.62
NC vs. AD	P	GMM+MS	89.1 (88.4)	0.97 (0.97)	0.92 (0.91)	0.86 (0.85)
		AAL	88.2	0.97	0.90	0.86
		t-test	89.1	0.97	0.92	0.85
	G	GMM+MS	87.7 (88.1)	0.96 (0.97)	0.93 (0.91)	0.81 (0.83)
		AAL	88.8	0.96	0.90	0.87
		t-test	87.1	0.95	0.93	0.79
NC vs. MCI	P	GMM+MS	63.2 (62.9)	0.72 (0.72)	0.65 (0.67)	0.61 (0.59)
		AAL	63.7	0.73	0.66	0.60
		t-test	67.1	0.75	0.68	0.65
	G	GMM+MS	64.6 (61.3)	0.74 (0.72)	0.66 (0.66)	0.64 (0.56)
		AAL	63.7	0.73	0.67	0.60
		t-test	65.8	0.72	0.66	0.65

“*” denotes the GMM+MS is significantly better than t-test approach at a statistical level of 0.05. The *p*-value is calculated by the corrected paired t-test tailored for comparing learning algorithms [41]). LIBSVM [40] is used to build the SVM models. A linear kernel is used, with a grid search for parameter optimization. Grid search considers only the optimization of the penalty parameter *C* in the linear SVM, selecting the value of *C* yielding the best classification result based on the training data. After the best value of *C* is found, we apply it to the test data. AUC: area under ROC curve. Each experiment was repeated 10 times with a 10-fold cross-validation. P: results using “primary sensorimotor cortex” region for intensity normalization. G: results using “grand mean” method for intensity normalization. Results from AIC are noted in brackets, following the BIC results.

**Table 5:** Result summary of three different methods on TUM dataset.

			Accuracy %	AUC	Sensitivity	Specificity
MCI vs. AD	P	GMM+MS	72.7 (74.0)	0.81 (0.80)	0.77 (0.78)	0.68 (0.69)
		AAL	74.8	0.80	0.77	0.72
		t-test	72.6	0.79	0.77	0.68
	G	GMM+MS	**73.8 (74.8)**	**0.82 (0.82)**	**0.77 (0.78)**	**0.71 (0.71)**
		AAL	70.5	0.78	0.72	0.69
		t-test	65.5	0.71	0.68	0.63
NC vs. AD	P	GMM+MS	90.5 (89.5)	0.93 (0.96)	0.94 (0.88)	0.89 (0.89)
		AAL	89.0	0.95	0.94	0.85
		t-test	89.4	0.97	0.86	0.90
	G	GMM+MS	91.6 (88.1)	0.97 (0.96)	0.94 (0.84)	0.90 (0.89)
		AAL	89.0	0.95	0.91	0.88
		t-test	92.0	0.98	0.93	0.91
NC vs. MCI	P	GMM+MS	88.0 (87.2)	0.95 (0.93)	0.87 (0.85)	0.89 (0.89)
		AAL	81.5	0.88	0.82	0.79
		t-test	81.1	0.90	0.64	0.89
	G	GMM+MS	87.7 (85.3)	0.95 (0.92)	0.87 (0.82)	0.88 (0.87)
		AAL	78.0	0.90	0.74	0.80
		t-test	89.5	0.94	0.89	0.90

Results from AIC are noted in brackets, following the BIC results.

**Fig 5:**
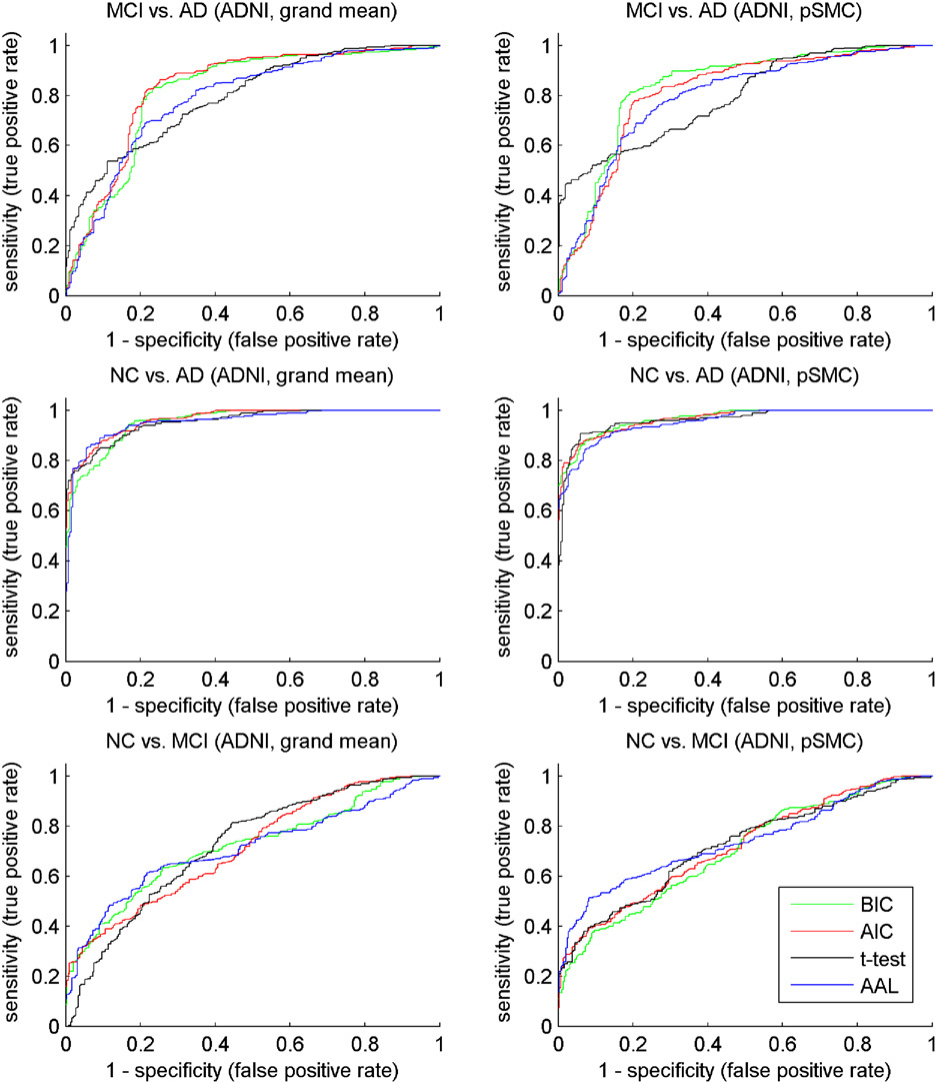
ROC curve of compared method on ADNI dataset. To plot the curve, we collected the predicted probabilities for all the test sets in 10 times 10-fold cross-validation, along with their true class labels. For the plotting of ROC curves, we refer to page 173 of the book by Witten et al. [42].

**Fig 6:**
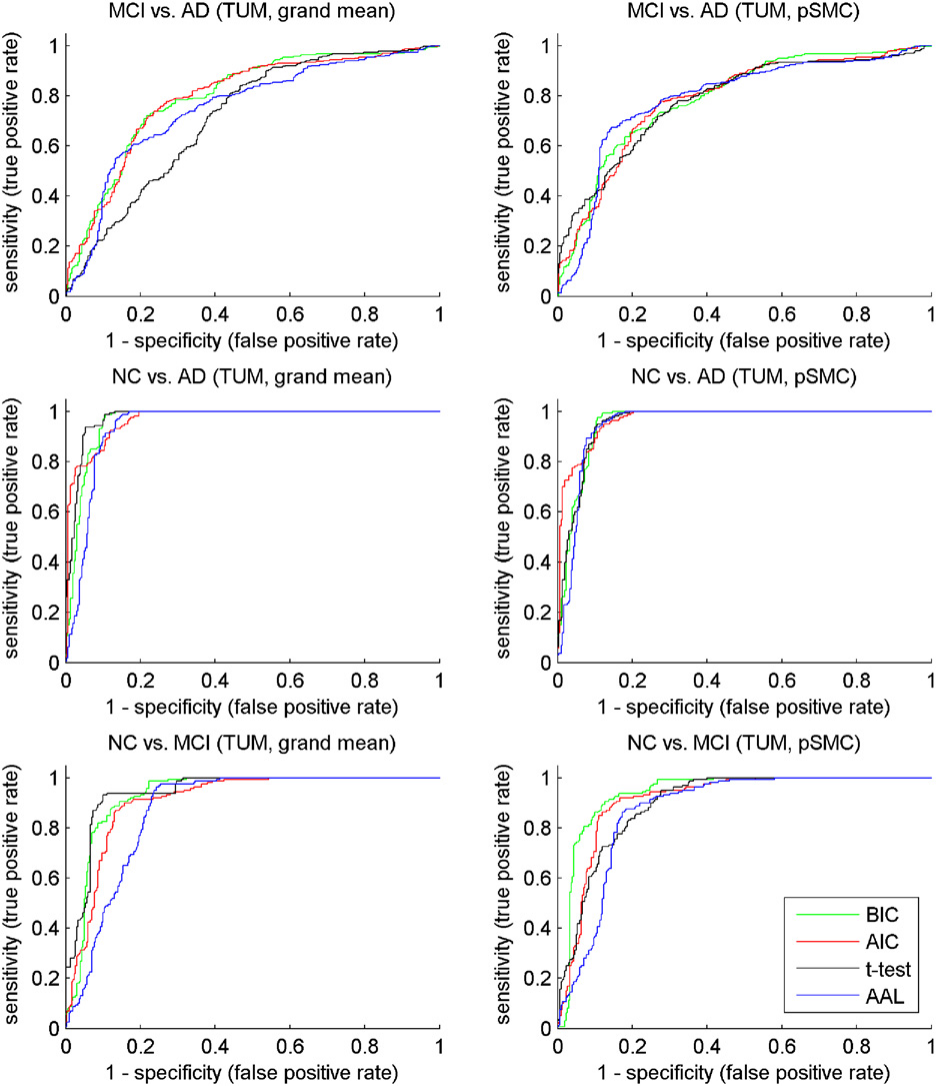
ROC curve of compared method on TUM dataset.

The ROC (receiver operating characteristic) curves shown in Figs 5 and 6 reveal the different performance of various methods, depicting the true positive rate against the false positive rate. The BIC (green) and AIC (red) curves cover a large portion of the t-test and AAL curves regarding MCI versus AD on both ADNI and TUM datasets. This observation complies with the results shown in Tables 4 and 5, i.e., the proposed method GMM+MS performs better than the compared ones in terms of MCI versus AD.

To sum up, the proposed method performs substantially better than the compared methods, in particular for MCI versus AD. Specifically, three comparisons out of four (TUM and ADNI datasets, grand mean and pSMC methods) demonstrate improved performance. A statistically significant result is also confirmed on the ADNI dataset. The limited NC sample size of 16 in the TUM dataset, as opposed to 30 in ADNI, may be one reason for less accurate results.

### Results Analysis

In general, the proposed method achieved either salient performance improvement or comparable results compared to more established methods using two different normalization methods in two independent datasets. However, the results are not perfectly consistent across the two datasets, which might be related to different types of scanners and different image acquisition methods, such as the amount of tracer used, whether an eye mask was used during the scan, and so forth.

**Fig 7:**
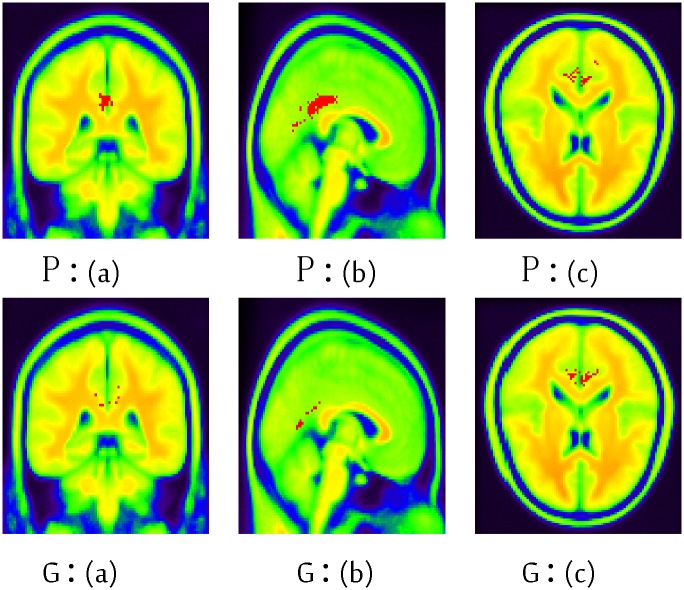
The informative regions (voxels) of MCI against AD using ADNI dataset of the 45th layer using BIC. (a): coronal view (b): sagittal view (c): transaxial view. P: primary sensorimotor cortex normalization; G: grand mean normalization. *x*, *y* and *z* are the width (91), depth (109) and height (91) respectively. The red points represent the informative voxels, whereas other colors are only used to depict the brain structure.

**Fig 8:**
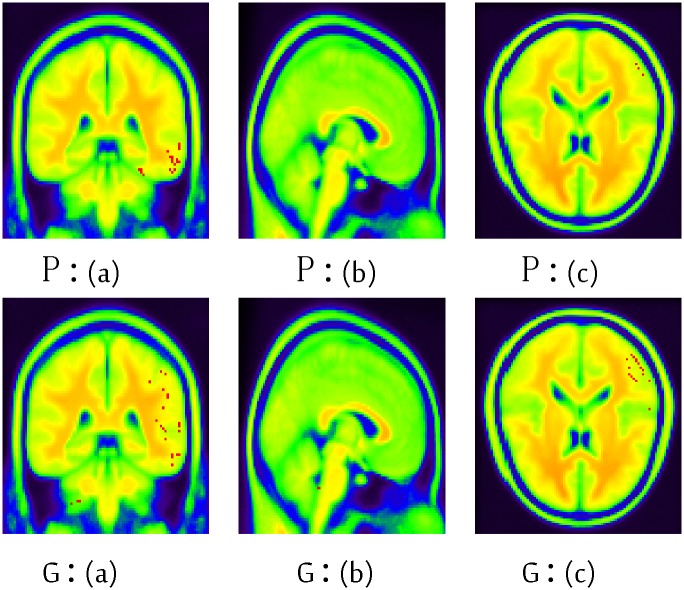
The informative regions (voxels) of MCI against AD using TUM dataset of the 45th layer using BIC. (a): coronal view (b): sagittal view (c): transaxial view. P: primary sensorimotor cortex normalization; G: grand mean normalization. *x*, *y* and *z* are the width (91), depth (109) and height (91) respectively. The red points represent the informative voxels, whereas other colors are only used to depict the brain structure.

The information in Tables 6 and 7 highlights the detailed brain region information regarding the contribution to the classification. These regions include areas which are typically involved in AD, such as the cingulum, precuneus and temporal regions. The red points in Figs 7 and 8 highlight these informative voxels (brain regions), which correspond to the information in Tables 6 and 7.

**Table 6:** Top-10 informative regions (voxels) of MCI against AD using ADNI dataset using BIC.

	Primary sensorimotor cortex normalization	Grand mean normalization
1	Precuneus_L: 20.4%	Precuneus_L: 28.4%
2	Cingulum_Post_L: 18.4%	Cingulum_Post_L: 21.1%
3	Precuneus_R: 17.9%	Precuneus_R: 14.2%
4	Cingulum_Mid_R: 15.7%	Cingulum_Post_R: 10.0%
5	Cingulum_Post_R: 10.0%	Calcarine_L: 5.50%
6	Cingulum_Mid_L: 5.74%	Cingulum_Mid_L: 5.28%
7	Cuneus_L:: 3.53%	Calcarine_R: 5.05%
8	Cerebellum_8_L: 2.87%	Cingulum_Mid_R: 4.36%
9	Calcarine_R: 2.06%	Cuneus_L: 3.90%
10	Occipital_Sup_L: 1.33%	Lingual_R: 1.15%

The top-ten clusters in GMM are recorded in each cross-validation, with different scores assigned to these clusters. The most informative cluster has the highest score. After 10 times 10-fold cross-validation, we rank these clusters according the overall score and select the top-ten, which are marked by the red points in Fig 7. Within these ten clusters, their corresponding AAL brain regions are identified and ranked according to the proportion denoted by the numbers in the Table. The region names remain as noted in the AAL template.

**Table 7:** Top-10 informative regions (voxels) of MCI against AD using TUM dataset using BIC.

	Primary sensorimotor cortex normalization	Grand mean normalization
1:	Temporal_Mid_L: 29.3%	Temporal_Mid_L: 40.8%
2:	Temporal_Inf_L: 29.0%	Temporal_Inf_L: 21.5%
3:	Fusiform_L: 13.0%	Occipital_Inf_L: 8.74%
4:	Occipital_Inf_L: 10.8%	Occipital_Mid_L: 8.65%
5:	Occipital_Mid_L: 6.47%	SupraMarginal_L: 4.42%
6:	Cerebelum_6_L: 3.41%	Parietal_Inf_L: 3.75%
7:	Parietal_Sup_L: 1.28%	Postcentral_L: 3.65%
8:	Angular_L: 1.28%	Fusiform_L: 2.11%
9:	Cerebelum_Crus1_L: 1.19%	Temporal_Sup_L: 1.83%
10:	Precuneus_L: 1.11%	Angular_L: 1.25%

After 10 times 10-fold cross-validation, we rank these clusters according the overall score and select the top-ten, which are marked by the red points in Fig 8. Within these ten clusters, their corresponding AAL brain regions are identified and ranked according to the proportion denoted by the numbers in the Table.

The use of two independent datasets is a major strength of our study, which keeps the research findings more objective as contrast to the other studies conducted on one dataset. In addition, two different intensity normalization methods, namely primary sensorimotor cortex and grand mean normalization, were applied to establish the experimental results. Tables 4 and 5 show that the proposed method is better compared to two other baseline methods.

### Error Analysis

To gain some insights into the classification difference between GMM+MS, AAL and the t-test methods, we exert to investigating the errors committed by the classifier. To be concise and illustrative, we take MCI vs. AD (grand mean normalization) in the ADNI dataset as a running example. Table 4 shows that the proposed method leads to an accuracy gain of 7%, which is a salient improvement. Looking at the misclassified images, we observe that a certain image is misclassified by AAL 4 times and 10 times by the t-test approach, but without any misclassification by GMM+MS. Therefore, this image, in fact it is an AD image, is selected for a more detailed study.

Recall that the SVM needs to compute the sign of *y* = *wx*+*b* to make a decision. Hence, knowing *w* and *b* is essential. Since *b* is only a constant, we omit it from further analysis. The studied AD image is regarded as the test data and all the rest is treated as training data. After training the model, the SVM returns the support vectors and the weights *w* (feature importance), such that the classification can be made upon *y* = *wx*+*b*. To stay illustrative and straightforward, we take a closer look at the Euclidean distance, since it gives a direct impression of dissimilarity. To this end, all the data, training and test, are multiplied by the weight vector *w*, such that each instance is re-weighted by their importance in terms of the SVM. Subsequently, the Euclidean distance is calculated between the test image and each training image (MCI and AD group). Finally, the mean weighted Euclidean distance is computed for the MCI and AD group, respectively, which represents the dissimilarity between the test image and the group. We compute the relative ratio to denote the dissimilarity:

$$\rho =\frac{D_{\text{MCI}}-D_{\text{AD}}}{D_{\text{AD}}},$$ (10)

where *D*_MCI_ is the mean distance between the test image and the MCI group, and the same as for *D*_*AD*_. The greater the *ρ*, the more similar to AD. As a result, *ρ*(GMM+MS) = 0.26, *ρ*(AAL) = 0.07, *ρ*(t-test) = 0.03. Hence, GMM+MS indicates the greatest value and thus it classifies this image correctly as AD. Therefore, the features derived from GMM+MS enable the SVM to make the correct decision in this case, in contrast to the AAL and t-test based methods.

## Discussion

In this paper, a machine learning approach, GMM+MS, is used to derive clusters based on an averaged NC PET image. The proposed method has the advantage of determining the number of clusters automatically, using a widely accepted model selection criterion. The model selection procedure assures that the derived model has a good trade-off between model fitting and model complexity. In such a way, a too complex model can be excluded, although it may have a good degree of model fitting. On the other hand, if a model is too concise, it may not have a satisfying level of model fitting. Therefore, the model selection procedure aims to keep the model complexity in a good balance. The two-phased algorithm first divides the voxel values into different bins and then applies the GMM+MS on the coordinate information at each of the bins to yield the final clusters. The resulting clusters have similar intensity values and are also geometrically connected.

The experimental results suggest that the proposed method can outperform the compared methods, in particular for discriminating MCI from AD. The underlying reason can be that the proposed algorithm is able to discover finer (smaller) clusters that are helpful in discriminating MCI from AD, while the AAL and t-test approach may fail to reveal such critical information. However, a little inconsistency is seen by the different intensity normalization methods, which also suggests that the intensity normalization procedure can be an important factor.

In the previous section, we also try to shed some light on the performance difference between these methods. Although the SVM is usually applied as a black-box classifier, we can still employ the support vectors and the weights to gain important insights. Since there are 150 features for the GMM+MS and t-test methods, and 232 for AAL, they are high-dimensional datasets, which makes it hard to analyze which features contribute to the correct classification in the end. However, by introducing the relative ratio computed from the Euclidean distance, it is possible to quantitatively show the difference between these approaches.

In terms of time complexity, the AAL method is the fastest because it is based on pre-defined brain regions. GMM+MS needs to work further based on defined AAL regions. In addition, the number of bins tested can also influence the running time. As for the t-test method, it is simple, but requires more memory to store the images for a group comparison, which can sometimes become a problem if there are too many images.

The proposed algorithm can be widely applied as a feature extraction method on medical imaging data, which can assist the medical imaging community to discover interesting discriminative brain voxels pattern. The applicability of the algorithm may reach broader application scenarios than merely AD classification, as long as imaging feature extraction is concerned. In particular, we also provide a thorough study on the comparison between AIC and BIC, which offers a clear guidance for the model selection issue.

One limitation of the work is the open problem of discriminating patients with MCI who progress to clinically diagnosable AD from those who remain clinically stable: this remains an important and challenging task. To deal with it, one may need a clearly defined dataset (MCI follow-up) and a reasonably sound algorithm, which is left for future work.

## Conclusions

The present work proposes a new clustering method, i.e., GMM+MS, for FDG-PET images. It has the advantage of determining the number of clusters automatically. This method is applied only on an NC image to define the clusters, and then the resulting clusters can be used to extract features from the MCI and AD images for automatic diagnosis. Throughout the experiments on two independent datasets, we not only demonstrate the merits of our method, but also show that the intensity normalization and different datasets (scanners) do play some role in the results. In conclusion, our results suggest that the data-driven extraction of informative brain regions may have a role to play in discriminating MCI from AD images for computer-aided diagnosis.

## Supporting Information

S1 File Txt A, Use of prediction model. It explains the use of prediction models of TUM data. Mat B, Prediction model of NC against AD using grand mean saved in MATLAB format. Represents the saved prediction model in MATLAB file format. The model is used for NC against AD, and is trained using the grand mean intensity normalization. The brain voxels are divided into 50 bins, which shows the best predictive performance by an internal cross-validation. BIC is used as the model selection method. In short, the model can be denoted as “predictionModel_grandMean_50_NCAD”. The meaning of the following models can be inferred similarly. Mat C, Prediction model of NC against MCI using grand mean saved in MATLAB format. Represents “predictionModel_grandMean_60_NCMCI”. Mat D, Prediction model of MCI against AD using pSMC saved in MATLAB format. Represents “predictionModel_PSMC_110_MCIAD”. Mat E, Prediction model of NC against MCI using pSMC saved in MATLAB format. Represents “predictionModel_PSMC_80_NCMCI”. Mat F, Prediction model of NC against AD using pSMC saved in MATLAB format. Represents “predictionModel_PSMC_50_NCAD”. Mat G, Prediction model of MCI against AD using grand mean saved in MATLAB format. Represents “predictionModel_grandMean_90_MCIAD”.
(ZIP)

## References

[1] ALZ. The prevalence of dementia worldwide, Alzheimer’s Disease International. 2008; http://www.alz.co.uk/adi/pdf/prevalence.pdf

[2] Drzezga A. Diagnosis of Alzheimer's disease with [18F] PET in mild and asymptomatic stages. Behavioural Neurology. 2009; 21: 101–15. 10.3233/BEN-2009-0228 19847049 PMC5444274

[3] Dubois B, Feldman HH, Jacova C, Dekosky ST, Barberger-Gateau P, et al Research criteria for the diagnosis of Alzheimer's disease: revising the NINCDS-ADRDA criteria. Lancet Neurology. 2007; 6: 734–746. 17616482 10.1016/S1474-4422(07)70178-3

[4] Górriz JM, Lassl A, Ramírez J, Salas-Gonzalez D, Puntonet CG, Lang EW. Automatic selection of ROIs infunctional imaging using Gaussian mixture models. Neuroscience Letters. 2009; 460: 108–111. 10.1016/j.neulet.2009.05.039 19454303

[5] Metz CE. Evaluation of CAD methods In Computer-Aided Diagnosis in Medical Imaging. In: Doi K.; MacMahon H.; Giger ML.; Hoffmann KL. 1999; 543–554.

[6] de Leon MJ, Mosconi L, Li J, De Santi S, Yao Y, et al Longitudinal CSF isoprostane and MRI atrophy in the progression to AD. Journal of Neurology. 2007; 254: 1666–1675. 17994313 10.1007/s00415-007-0610-z

[7] Fjell AM, Walhovd KB, Fennema-Notestine C, McEvoy LK, Hagler DJ, et al CSF biomarkers in prediction of cerebral and clinical change in mild cognitive impairment and Alzheimer's disease. Journal of Neuroscience. 2010; 30: 2088–2101. 10.1523/JNEUROSCI.3785-09.2010 20147537 PMC2828879

[8] McEvoy LK, Fennema-Notestine C, Roddey JC, Hagler DJ Jr, Holland D, et al Alzheimer disease: quantitative structural neuroimaging for detection and prediction of clinical and structural changes in mild cognitive impairment. Radiology. 2009; 251: 195–205. 10.1148/radiol.2511080924 19201945 PMC2663582

[9] Cuingnet R, Gerardin E, Tessieras J, Auzias G, Lehericy S, et al Automatic classification of patients with Alzheimer’s disease from structural MRI: A comparison of ten methods using the ADNI database. NeuroImage. 2011; 56: 766–781. 10.1016/j.neuroimage.2010.06.013 20542124

[10] Liu MH, Zhang DQ, Shen D.G. Ensemble sparse classification of Alzheimer's disease. NeuroImage. 2012; 60: 1106–1116. 10.1016/j.neuroimage.2012.01.055 22270352 PMC3303950

[11] Gray KR, Aljabar P, Heckemann RA, Hammers A, Rueckert D. Rand forest-based similarity measures for multi-modal classification ofAlzheimer's disease. NeuroImage. 2013; 65: 167–175. 10.1016/j.neuroimage.2012.09.065 23041336 PMC3516432

[12] Li R, Hapfelmeier A, Schmidt J, Perneczky R, Drzezga A, Kurz A, Kramer S. A Case Study of Stacked Multi-view Learning in Dementia Research Proceedings of the 13th Conference on Artificial Intelligence in Medicine. 2010; 60–69, Berlin, Heidelberg, Springer LNCS.

[13] Zhang DQ, Wang YP, Zhou LP, Yuan H, Shen DG. Multimodal classification of Alzheimer's disease and mild cognitive impairment. NeuroImage. 2011; 55: 856–867. 10.1016/j.neuroimage.2011.01.008 21236349 PMC3057360

[14] Sharif MS, Abbod M, Amira A, Zaidi H. Artificial Neural Network-Statistical Approach for PET Volume Analysis and Classification Advances in Fuzzy Systems—Special issue on Hybrid Biomedical Intelligent Systems. 2012; Hindawi.

[15] Salaun PY, Campion L, Ansquer C, Frampas E, Mathieu C, et al 18F-FDG PET predicts survival after pretargeted radioimmunotherapy in patients with progressive metastatic medullary thyroid carcinoma. European Journal of Nuclear Medicine and Molecular Imaging. 2014; 41: 1501–1510. 10.1007/s00259-014-2772-0 24806110

[16] Echeverria AE, McCurdy M, Castillo R, Bernard V, Ramos NV, et al Proton therapy radiation pneumonitis local dose–response in esophagus cancer patients. Radiotherapy and Oncology. 2013; 106: 124–129. 10.1016/j.radonc.2012.09.003 23127772 PMC3696882

[17] Lu L, Karakatsanis NA, Tang J, Chen W, Rahmim A. 3.5D dynamic PET image reconstruction incorporating kinetics-based clusters. Physics in Medicine and Biology. 2012; 57: 5035–5055. 10.1088/0031-9155/57/15/5035 22805318 PMC3445711

[18] Price JC, Klunk WE, Lopresti BJ, Lu XL, Hoge JA. Kinetic modeling of amyloid binding in humans using PET imaging and Pittsburgh Compound-B. Journal of Cerebral Blood Flow & Metabolism. 2005; 25: 1528–1547. 15944649 10.1038/sj.jcbfm.9600146

[19] Statistical Parametric Mapping. 2005. http://www.fil.ion.ucl.ac.uk/spm/software/spm5/

[20] Lopez M, Ramirez J, Gorriz JM, Salas-Gonzalez D, Alvarez I, Segovia F, Puntonet C. Automatic tool for Alzheimer’s disease diagnosis using PCA and Bayesian classification rules. IET Electronics Letters. 2009; 45: 389–391.

[21] Nobili F, Salmaso D, Morbelli S, Girtler N, Piccardo A, et al Principal component analysis of FDG PET in amnestic MCI. European Journal of Nuclear Medicine and Molecular Imaging. 2008; 35: 2191–2202. 10.1007/s00259-008-0869-z 18648805

[22] Górriz JM, Segovia F, Ramírez J, Lassl A, Salas-Gonzalez D. GMM based SPECT image classification for the diagnosis of Alzheimer’s disease. Applied Soft Computing. 2011; 11: 2313–2325.

[23] Segovia F, Górriz JM, Ramirez J, Salas-Gonzalez D, Alvarez I, Lopez M, Chaves R. A comparative study of feature extraction methods for the diagnosis of Alzheimer’s disease using the ADNI database. Neurocomputing. 2012; 75: 64–71.

[24] Tzourio-Mazoyer N, Landeau B, Papathanassiou D, Crivello F, Etard O, et al Automated anatomical labeling of activations in SPM using a macroscopic anatomical parcellation of the MNI MRI single-subject brain. NeuroImage. 2002; 15: 273–289. 11771995 10.1006/nimg.2001.0978

[25] Yakushev I, Landvogt C, Buchholz HG, Fellgiebel A, Hammers A, et al Chose of reference area in studies of Alzheimer’s disease using positron emission tomography with fluorodeoxyglucose-F18. Psychiatry Research. Neuroimaging. 2008; 164: 143–153. 10.1016/j.pscychresns.2007.11.004 18930634

[26] Bishop CM. Pattern Recognition and Machine Learning. 2006 Springer.

[27] Dempster AP, Laird NM, Rubin DB. Maximum Likelihood from Incomplete Data via the EM Algorithm. Journal of the Royal Statistical Society. Series B (Methodological). 1977; 39: 1–38.

[28] Schwarz GE. Estimating the dimension of a model. Annals of Statistics. 1978; 6: 461–464.

[29] Akaike H. A new look at the statistical model identification. IEEE Transactions on Automatic Control. 1974; 19: 716–723.

[30] Murphy KP. Machine Learning: A Probabilistic Perspective. 2012 The MIT Press.

[31] Degroote P, Briquet M, Catala C, Uytterhoeven K, Lefever K, et al Evidence for nonlinear resonant mode coupling in the Beta Cep star HD 180642 (V1449 Aql) from CoRoT space- based photometry. Astronomy & Astrophysics (A&A). 2009; 506: 111–123.

[32] Preacher KJ, Zhang GJ, Kim CT, Mels G. Choosing the Optimal Number of Factors in Exploratory Factor Analysis: A Model Selection Perspective. Multivariate Behaviora Research. 2013; 48: 28–56. 10.1080/00273171.2012.710386 26789208

[33] Burnham KP, Anderson DR. Multimodel Inference Understanding AIC and BIC in Model Selection. Sociological Methods & Research. 2004; 33: 261–304.

[34] Naik PA, Shi P, Tsai CL. Extending the Akaike Information Criterion to Mixture Regression Models, Journal of the American Statistical Association. 2007; 102: 244–254.

[35] Kass RE, Raftery AE. Bayes Factors. Journal of the American Statistical Association. 1995; 90: 773–795.

[36] Chen YW, Lin CJ. Combining SVMs with various feature selection strategies In: Feature Extraction. Foundations and Applications. 2006 Springer.

[37] Smialowski P, Fishman D, Kramer S. Pitfalls of supervised feature selection. Bioinformatics. 2010; 26: 440–443. 10.1093/bioinformatics/btp621 19880370 PMC2815655

[38] Burges CJC. A tutorial on support vector machines for pattern recognition. Data Mining and Knowledge Discovery. 1998; 2: 121–167.

[39] Chu C, Hsu AL, Chou KH, Bandettini P, Lin CP. Does feature selection improve classification accuracy? Impact of sample size and feature selection on classification using anatomical magnetic resonance images. NeuroImage. 2012; 60: 59–70. 10.1016/j.neuroimage.2011.11.066 22166797

[40] Chang CC, Lin CJ. LIBSVM: A library for support vector machines. ACM Transactions on Intelligent Systems and Technology. 2011; 2: 1–27.

[41] Bouckaert R, Frank E. Evaluating the replicability of significance tests for comparing learning algorithms, The 8th Pacific-Asia Conference on Knowledge Discovery and Data Mining (PAKDD). 2004; 3–12. Springer.

[42] Witten IH, Frank E, Hall MA. Data Mining: Practical Machine Learning Tools and Techniques, Third Edition 2011 The Morgan Kaufmann Series in Data Management System.

